# Speech in noise perception improved by training fine auditory discrimination: far and applicable transfer of perceptual learning

**DOI:** 10.1038/s41598-020-76295-9

**Published:** 2020-11-09

**Authors:** Xiang Gao, Tingting Yan, Ting Huang, Xiaoli Li, Yu-Xuan Zhang

**Affiliations:** State Key Laboratory of Cognitive Neuroscience and Learning, IDG/McGovern Institute for Brain Research, Beijing Normal University, Beijing, 100875 China

**Keywords:** Perception, Perception, Learning and memory, Learning and memory

## Abstract

A longstanding focus of perceptual learning research is learning specificity, the difficulty for learning to transfer to tasks and situations beyond the training setting. Previous studies have focused on promoting transfer across stimuli, such as from one sound frequency to another. Here we examined whether learning could transfer across tasks, particularly from fine discrimination of sound features to speech perception in noise, one of the most frequently encountered perceptual challenges in real life. Separate groups of normal-hearing listeners were trained on auditory interaural level difference (ILD) discrimination, interaural time difference (ITD) discrimination, and fundamental frequency (F_0_) discrimination with non-speech stimuli delivered through headphones. While ITD training led to no improvement, both ILD and F_0_ training produced learning as well as transfer to speech-in-noise perception when noise differed from speech in the trained feature. These training benefits did not require similarity of task or stimuli between training and application settings, construing far and wide transfer. Thus, notwithstanding task specificity among basic perceptual skills such as discrimination of different sound features, auditory learning appears readily transferable between these skills and their “upstream” tasks utilizing them, providing an effective approach to improving performance in challenging situations or challenged populations.

## Introduction

To extract target information from a competing and intervening background environment, such as speech perception in noise, is a major perceptual challenge that people encounter daily. Improving perception in such situations is of great interest in rehabilitative, professional, and educational settings. However, the benefit of perceptual learning is often bound to the training material and task^for review,^ ^1,2^. For example, training word recognition in noise with one word set failed to improve performance with another set^3^, and training discrimination of one sound feature did not transfer to discrimination of another feature even with the same sound^4^. The past decade has seen vigorous research and considerable progress on understanding and overcoming learning specificity^5–8^. To date, such research has primarily focused on stimulus specificity due to both practical and theoretical concerns. Theoretically, stimulus specificity of learning has often been linked to stimulus selectivity of neural responses along the sensory processing hierarchy to shed light onto learning loci^1,8^. Practically, stimulus specificity is the foremost limit of learning utility. As different perceptual tasks and their situations of application typically involve different stimuli, across-task transfer of learning appears, if not impossible, at least impractical before stimulus specificity can be resolved. Therefore, though nearly all basic perceptual skills can improve with training, whether training these skills can benefit real-life perceptual challenges such as speech recognition in noise has rarely been examined. Here, we propose and confirm that, notwithstanding learning specificity of and among the basic skills, such benefits can be attained.

We started with a simple assumption that any perceptual performance, may it be as simple as deciding if two pure tones are the same or as complicated as speech comprehension at a cocktail party, would depend on a hierarchical network of sensory, perceptual, cognitive, and affective processes, in which processes at similar levels of the hierarchy such as extraction of different stimulus features can function in parallel while those at different levels are serially organized^5^. According to this network view, specificity of learning reflects presence of parallel processing at the learning level: stimulus specificity arises when different stimuli are processed separately for the training task, and task specificity arises when the transfer task relies on a process parallel to the learned one at that level of processing. This account, while concurring with most current theories of learning in terms of stimulus specificity, has generated a contrasting prediction regarding task specificity: learning should transfer to other tasks that engage the trained component process. Supporting this prediction, we have shown that learning can transfer between perceptual (tone frequency discrimination) and cognitive (n-back) tasks^9^, which presumably share critical memory processes. A more direct test of the prediction is whether training basic perceptual skills should benefit “up-stream” tasks employing those skills, with the trained skills themselves serving as shared processes. Towards this end, we examined whether speech perception in noise, one of the most frequently encountered real-life perceptual challenges, could benefit from training fine discrimination of sound features useful for signal–noise separation with non-speech stimuli.

Among the sound features that can contribute to signal–noise separation, the most studied ones are cues to sound source location, primarily interaural time differences (ITDs) and interaural level differences (ILDs). These cues, among others, are used to reduce the masking effect of noise originating from sources spatially separate from the target sound, a phenomenon known as spatial release from masking, ^e.g.,^ ^10,11^ though in the case of ILDs, it has been argued that the masking release could be to certain extent attributed to “better-ear listening”, i.e., listening to the ear with better signal-to-noise ratios^12–15^. Similarly, noise masking can be reduced with spectral or temporal cues. For example, fundamental frequency (F_0_) is used to separate speech of different voices (speakers), attenuating speech-to-speech masking^16^. F_0_ perception under some conditions, such as with “unresolved” tones that cannot be separated by auditory filters at the peripheral auditory system, relies on processing of “temporal fine structure”^17^, a temporal skill important for speech perception in noise with amplitude fluctuations^18–20^. Human discrimination of ILDs^21,22^, ITDs^23,24^, and F_0_^25^ has been demonstrated, to various extent, to improve with training. Thus, these cues were chosen to test whether learning of basic auditory skills can transfer, across task and stimulus differences, to speech perception in noise.

## Methods and materials

### Participants and equipment

A total of 83 young healthy adults (54 females, mean age 21.9 ± 2.5 years) participated in the experiment. They were recruited from Beijing Normal University campus, and gave written consent for participation. All of the participants had normal hearing (tone threshold ≤ 20 dB HL from 0.25 to 8 kHz at each ear) and no previous experiences with psychoacoustic studies. The experimental procedure was approved by the Beijing Normal University Research Committee. The study was carried out in accordance with relevant guidelines and regulations. All participants provided informed consent.

Testing and training were conducted in a double-walled sound attenuating booth using custom computer programs based on the Psychtoolbox for Matlab^26,27^. Auditory stimuli were digitally generated. The sampling rate was 192 kHz to increase time resolution close to 5 μs when interaural time difference (ITD) was manipulated and was 44.1 kHz otherwise. Speech stimuli were manipulated with Praat^28^ for duration and pitch adjustments. Sounds were presented binaurally via circumaural headphones (Sennheiser HD-650).

### Experimental design

The study consisted of three training experiments, two training spatial skills (interaural level difference, or ILD, and interaural time difference, or ITD, discrimination), and one training spectral skills (fundamental frequency, or F0 discrimination). A pretest-training-posttest design was used for all experiments. Training involved repetitive practice on a single auditory task approximately half an hour per day for six to seven consecutive days except for weekends. In the pre- and posttests, the trained group, together with an untrained control group were tested on the training task as well as a speech-in-noise task. Details of training procedure and testing tasks are described in the following sections.

### Tasks and stimuli

#### Auditory discrimination tasks

For the three training tasks, ILD, ITD, and F0 discrimination, performance was measured and trained with a two- (ITD and ILD) or three- (F0) interval, forced-choice procedure and adaptive staircases. Each staircase consisted of 60 trials (a block), beginning with a lead-in phase in which the discrimination signal was increased after each incorrect response and decreased after each correct response. The point at which the direction of signal change switched from increasing to decreasing or from decreasing to increasing was denoted as a reversal. After the third reversal, the adaptive rule switched to 3-down-1-up (ITD and ILD) or 2-down-1-up (F0) to estimate discrimination threshold corresponding to 79% (ITD and ILD) or 71% (F0) correct performance on the psychometric function^29^. A visual feedback was provided after each response.

##### Interaural level difference (ILD) discrimination

On each trial, two 300-ms (including 10-ms rise/fall raised cosine ramps) sounds differing only in ILD value were presented binaurally with a 500-ms silence gap in between. The sounds were Gaussian noise lowpass filtered at 1 kHz sinusoidally amplitude modulated at 8 Hz with no interaural time or phase differences. Amplitude modulation has been shown to enhance across-stimulus transfer of ILD discrimination learning^22^. The low-frequency region was chosen because it affords greater share of speech energy and produced effective ILD learning in a pilot experiment. Listeners were instructed to report whether the second sound was to the left or right of the first sound by pressing the left or right arrow key on a computer keyboard. ILD difference between the two sounds (ΔILD) served as the discrimination signal. For each block, ΔILD started at 6 dB for each block and was adaptively changed with a step size of 0.8 dB in the lead-in phase and 0.2 dB thereafter. The ILD value was fixed in one of the two sounds randomly selected at each trial, referred to as the standard ILD. ILD in the other sound was the standard ILD plus ΔILD. Each sound was presented at the left ear at 70 dB SPL minus 0.5 times the desired ILD, and at the right ear at 70 dB SPL plus 0.5 times the desired ILD.

Participants were instructed to attend to the sound image inside their head and indicate the sound that was lateralized further to their right ear. Though discouraged, ILD discrimination could be performed by listening to sound level change at a single ear (level difference = ΔILD/2) while ignoring input from the other. Possible implications of this alternative strategy will be elaborated in Discussion.

ILD training consisted of 6 to 7 daily sessions, 12 blocks per session, of ILD discrimination with a standard ILD of 0 dB (perceived at around the midline of the head). During pre- and posttests, both the training condition and an untrained condition with a standard ILD of 6 dB were tested for 2 blocks per condition.

##### Interaural time difference (ITD) discrimination

On each trial, two 300-ms (including 10-ms rise/fall raised cosine ramps) 1-kHz lowpass Gaussian noise with a 500-ms inter-stimulus interval were presented binaurally at 70 dB SPL. The two sounds differed only in their ongoing ITD. This difference (ΔITD) served as discrimination signal. Task instruction was the same as the ILD task. Each sound was gated on and off simultaneously at the two ears. Ongoing ITDs were set by playing to the two ears two 300-ms sections of a slightly longer noise sample, the onsets of which were separated by the desirable ITD. Discrimination was conducted around a nominal standard ITD: At each trial, ITD in one sound was standard ITD plus 0.5 times ΔITD, and in the other was standard ITD minus 0.5 times ΔITD. The presentation order was randomized across trials. ΔITD started at 500 μs for each staircase, and was adaptively varied on the logarithmic scale^30^. The step size was 2 during the lead-in phase, and was 1.41 thereafter. Threshold estimation and subsequent analyses were also conducted on the logarithmic scale.

ITD training consisted of 7 daily sessions of 12 blocks with a nominal standard ITD of 0 μs. During pre- and posttests, both the training condition and an untrained condition with a nominal standard ITD of 150 μs were tested for 2 blocks per condition.

##### Fundamental frequency (F_0_) discrimination

The F0 task was modified after two previous studies on F0 discrimination training^31,32^. Each trial consisted of three 200-ms harmonic complexes (with 10-ms rise/fall ramps) separated by 300-ms inter-stimulus intervals presented within a pink noise background that started 300 ms earlier and ended 300 ms later than the complex tones. Two of the complexes were identical (the standard), and the third, randomly selected at each trial, had a higher F0. The F0 difference (ΔF0) served as discrimination signal. Listeners were instructed to indicate which sound was different from the others by pressing a key on the keyboard. Each complex tone was generated by adding in sine (0°) phase the 5th to 27th harmonics of the desirable F0 and bandpass filtered the stimulus between the 10th and the 20th order of the standard F0 (e.g., between 2 to 4 kHz for a standard F0 of 200 Hz). Relatively high-order harmonics were used because compared to lower-order ones, they appeared to generate less specific learning^33^. The filter had a flat top and a slope of 80 dB/octave. The same filter was applied to all of the three complex tones at each trial. The background noise was intercepted from a 10-s pink noise generated offline with a 6 dB/octave slope and presented with an overall level of 55 dB SPL. The complex tones were presented at 65 dB SPL. Within each block, standard F0 was roved between 120 and 240 Hz, with the constraint that variation between consecutive trials should be between 5 and 30 Hz. Standard roving has been shown to enhance magnitude and transferability of frequency learning^9,34^. ΔF0 started at 50% and was adaptively adjusted on the logarithmic scale. Similar to the ITD task, the step size was 2 during the lead-in phase and was 1.41 thereafter. All subsequent calculations were also conducted on the logarithmic scale.

The roving condition was used for both training and testing. Training consisted of 7 daily sessions of 12 blocks, while 3 blocks were conducted in each of the pre- and posttests.

#### Speech perception in noise

Speech perception in noise was measured using word identification in the ILD and ITD training experiments and vowel identification in the F0 training experiment.

##### Word identification

At each trial, a monosyllable Chinese word spoken by a native male voice was presented within a noise masker. Different stimulus sets were used in the pre- and posttests. Each stimulus set was comprised of 16 syllables each with 4 variations in lexical tone, resulting in a one-interval, 64-alternative forced choice task. The choice options were displayed on the computer screen, with a 4 × 4 grid containing the Chinese spelling (Pinyin) of the 16 syllables flanked on the right by a 4 × 1 grid containing the digits (1 to 4) denoting the lexical tones. Listeners were instructed to indicate the perceived syllable and tone by mouse clicks. There was no trial-by-trial feedback, but overall performance in percent correct was visually displayed upon finishing a block. All of the speech tokens were presented at a constant level of 65 dB SPL, and at their originally recorded durations (340 to 780 ms long, 539 ms on average). The masker was Gaussian noise filtered to match the long-term spectrum of spoken Chinese characters, gated on and off simultaneously with the speech stimuli. Noise was presented at four signal-to-noise ratios (SNRs), − 12, − 9, − 6, and − 3 dB. The SNRs were determined based on a pilot study to cover the major portion of performance range in most listeners.

To examine the use of spatial skills, the task was conducted under two spatial configurations. While the target speech stimuli were always presented diotically (perceived approximately in the middle of the head), the noise maker was either co-located with or spatially separated from the target. In the co-located condition, the masker was also diotic, with both ILD and ITD set to zero. In the separated condition, the masker was lateralized to the right by ILD (6 dB, by increasing sound level at right ear and decreasing sound level at left ear by 3 dB) in the ILD training experiment and by ITD (150 μs) in the ITD training experiment.

Word identification was assessed in each of the pre- and posttests with 80 trials (20 trials per SNR mixed in random order) for each spatial condition. The order of spatial conditions was randomized across listeners but maintained for each listener through the tests. In case dramatic improvement resulted from testing and masked effect of spatial training, in the ILD training experiment, the word identification task was ‘pre-trained’ for six blocks before the pretest for all groups. Because improvement caused by such pre-training was moderate (3.9 ± 10% in identification accuracy), in the following ITD and F0 training experiments, no pre-training was provided for the speech task.

##### Vowel identification task

At each trial, a 350-ms monophthong Chinese vowel (the target) embedded in the middle of a 1000-ms clip of babble noise (the masker) was presented binaurally. Listeners were instructed to select the perceived vowel from a 2 × 3 grid labelling in Pinyin all of the Mandarin Chinese vowels (a, o, e, i, u, ü). The six vowels were presented equally frequently but in randomized order. All of the vowels were pronounced in tone 1. The level of target stimuli was fixed at 65 dB SPL, and the level of noise masker was varied to produce SNRs of − 13, − 9, − 5, − 1, and 3 dB.

This task tapped into the ability to take advantage of pitch-related spectral and temporal skills for hearing in noise. The noise masker was generated by mixing six sound tracks of 10-s random words spoken by six different male talkers. The multi-talker babble masker has been shown to produce greater masking for phoneme identification than steady-state noise and single-talker competing speech^35,36^. F_0_s of the six talkers were adjusted to distribute evenly between 87 and 161 Hz. The target vowels were spoken either by a male talker with an F_0_ in the middle of those of the babble noise (124 Hz), or by a female talker with an F_0_ 10 semitones higher (229 Hz).

In each of the pre- and posttests, listeners completed three blocks of 60 trials, with SNRs and target talker conditions randomized. Before the test, listeners practiced another block of 60 trials, half with and half without the babble noise, to familiarize themselves with the task and the target talkers’ voices.

#### Auditory working memory (WM) task

A Tone n-back task was used to access and train auditory WM^9^. At each trial, a sequence of 40 + n pure tones was presented at the rate of 2.5 s/item. A tone matching that presented n positions back was denoted as a target and there were twelve targets randomly distributed in each sequence. Before and during each trial, n was displayed on the screen. Listeners were instructed to indicate a target by pressing a key and to make no response for non-targets. Visual feedback was provided after each response and upon finishing a sequence. All tones were 100-ms long (including 10-ms raised cosine ramps) and presented at 60 dB SPL. There were eight sets of eight tone frequencies selected from the range of 1080 to 4022 Hz, with neighboring frequencies in each set separated by at least one equivalent rectangular bandwidth (ERB) so that they were clearly distinguishable from each other. WM performance was indexed by d’, calculated as Z(hit rate) – Z(false alarm rate), where Z is the inverse cumulative Gaussian distribution.

WM training was used as active control for F0 training and similar to F0 training, consisted of 7 daily sessions of approximately half an hour of practice per session. To enable learning, WM training started with 2-back and switched to 3-back after three sessions^9^. Twelve sequences were completed in each training session and two sequences were completed in each of the pre- and posttests.

## Results

### Training spatial skills

**Figure 1:**
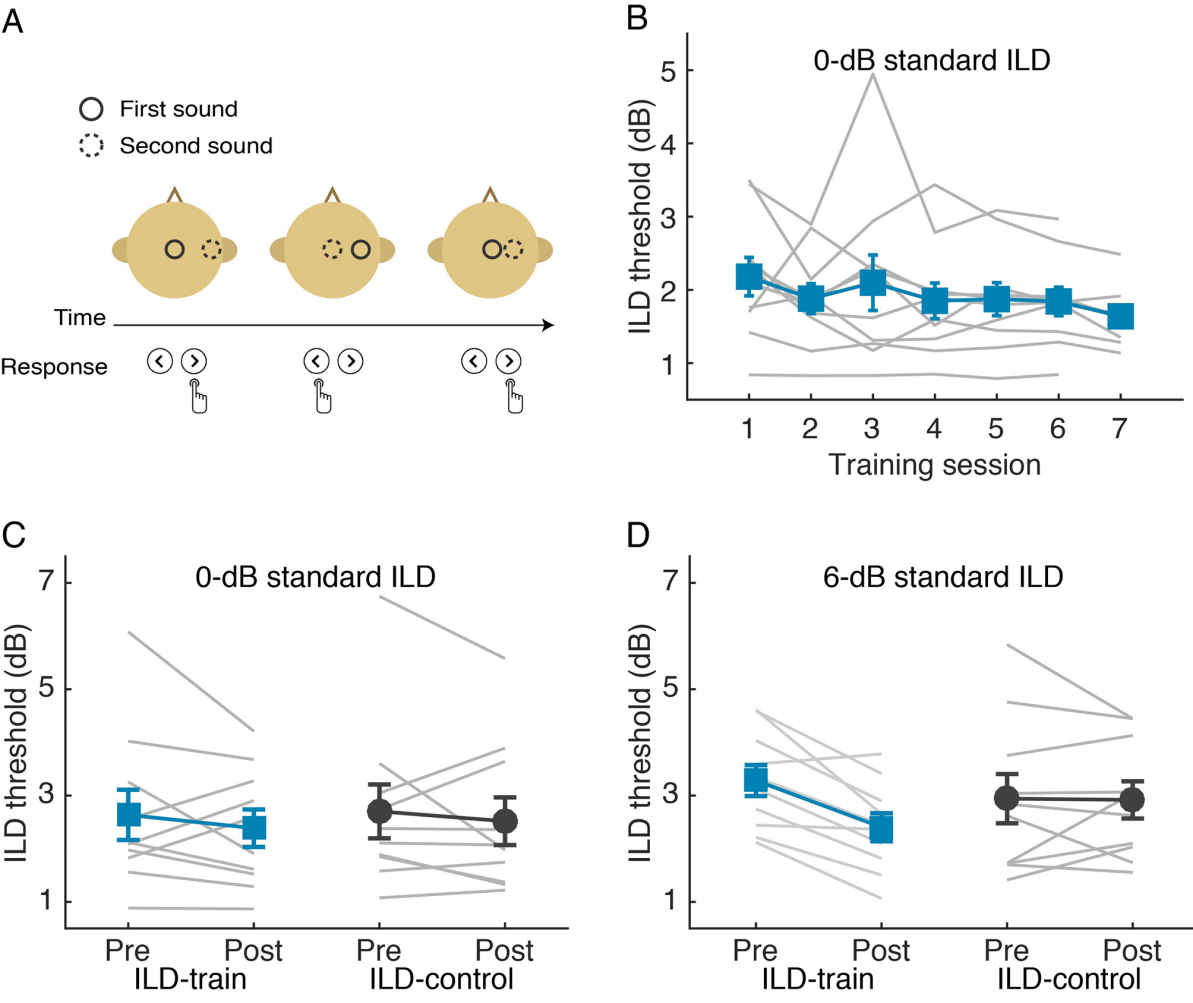
ILD discrimination training. **(A)** Illustration of ILD discrimination task, shown in 3 trials. **(B–D)** Individual (grey lines) and group mean (filled symbols) ILD discrimination thresholds through training sessions **(B)** and between the pre- and post-training tests for the training condition **(C)** and an untrained condition with 6-dB standard ILD **(D)**. Error bars in all figures stand for one S.E.M.

We first examined whether training spatial discrimination could improve speech perception in noise. Healthy young adults practiced on discrimination of one of the two sound localization cues, interaural level and time differences (ILDs and ITDs), for six to seven daily 35-min sessions. During training, the listeners were instructed to indicate direction of changes in perceived sound location (Fig. 1A) caused by changes in either ILD (N = 10) or ITD (N = 12). Before and after training, the training groups, together with their respective no-training control groups (ILD-control: N = 10; ITD-control: N = 12), were tested on a Mandarin word-in-noise recognition task as well as the respective training task.

#### ILD training

ILD discrimination (Fig. 1A) was trained with a noise low-passed at 1 kHz sinusoidally amplitude modulated (AM) at 8 Hz, with a standard location of 0-dB ILD (the midline). The low-pass AM noise was chosen because the low-frequency region contains most energy in speech stimuli and amplitude modulation has been shown to enhance transfer of ILD learning^22^.

ILD discrimination threshold decreased with training (Fig. 1B; linear regression: F_1,5_ = 10.88, p = 0.022, adjusted R^2^ = 0.622). However, compared to the ILD-control group (N = 10), the ILD-train group did not improve more on the trained condition (Fig. 1C; repeated measure ANOVA, group by test interaction: F_1, 18_ = 0.03, p = 0.865, partial η^2^ = 0.002; group effect: F_1, 18_ = 0.027, p = 0.872, partial η^2^ = 0.001; test effect: F_1, 18_ = 1.31, p = 0.267, partial η^2^ = 0.068). Instead, they improved more on an untrained condition of the training task, where the standard sound location was 6-dB instead of 0-dB ILD (Fig. 1D; group by test interaction: F_1, 18_ = 7.78, p = 0.012, partial η^2^ = 0.302; group effect: F_1, 18_ = 0.34, p = 0.856, partial η^2^ = 0.002; test effect: F_1, 18_ = 8.67, p = 0.009, partial η^2^ = 0.325). Compared to the trained location, ILD discrimination threshold at the untrained location was significantly higher before training (rmANOVA, effect of condition: F_1, 9_ = 5.63, p = 0.042, partial η^2^ = 0.385), but not after (F_1, 9_ = 0.006, p = 0.942, partial η^2^ = 0.001).

**Figure 2:**
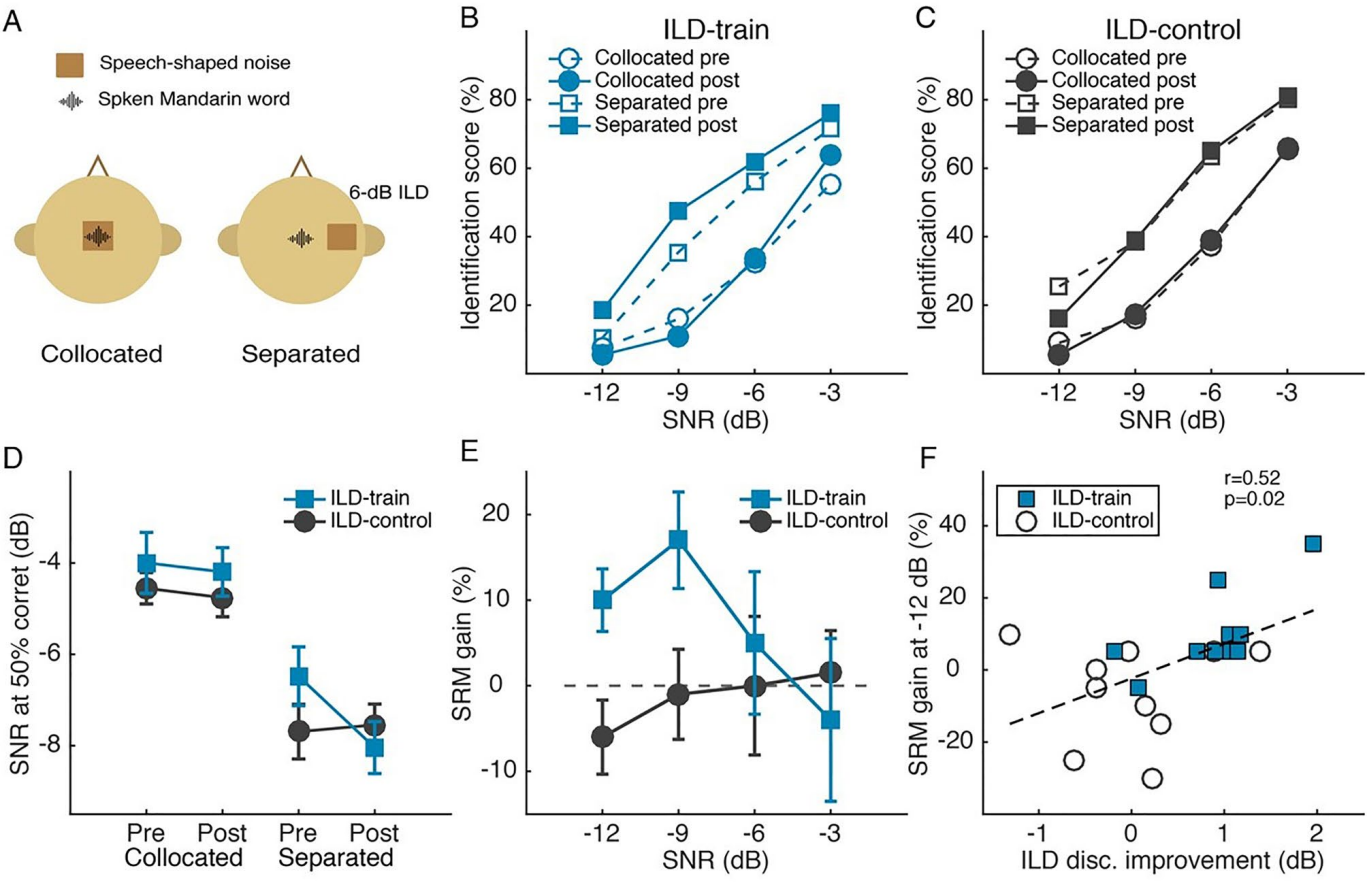
Effect of ILD discrimination training on sech perception in noise. **(A)** Illustration of the Mandarin word-in-noise task. **(B,C)** Mandarin word identification score (in % correct) across SNR levels for the ILD-train **(B)** and ILD-control **(C)** groups. **(D)** Speech reception threshold (SNR at 50% correct identification). **(E)** Pre-to-posttest gain in spatial release from masking (SRM). **(F)** Correlation between ILD learning at the 6-dB ILD condition and SRM gain at − 12-dB SNR.

Speech perception was measured by Mandarin word identification (Fig. 2A) in collocated and spatially separated (by 6-dB ILD) long-term speech shaped noise. The task was pre-trained before the pretest to allow for rapid learning of the speech task, in case such learning should confound with the ILD training effect.

At the pretest, speech perception performance did not differ between groups (Fig. 2B,C; rmANOVA, effect of group: F_1, 18_ = 2.81, p = 0.111, partial η^2^ = 0.135), but differed markedly between the collocated and spatially separated noise conditions (effect of condition: F_1, 18_ = 63.00, p < 0.001, partial η^2^ = 0.778), indicating that the 6-dB ILD difference successfully produced spatial release from masking (SRM). Between the pre- and posttests, word identification score (in percent correct) of the ILD-control group did not change for either spatially separated (Fig. 2C; rmANOVA, effect of test: F_1, 9_ = 0.987, p = 0.346, partial η^2^ = 0.099) or collocated (F_1, 9_ = 0.018, p = 0.896, partial η^2^ = 0.002) noise configuration, indicating that word identification performance was successfully stabilized by the pre-training. Critically, the ILD-train group improved significantly for spatially separated (Fig. 2B; rmANOVA, effect of test: F_1, 9_ = 12.94, p = 0.006, partial η^2^ = 0.590), but not for collocated (F_1, 9_ = 0.05, p = 0.829, partial η^2^ = 0.005) noise, consistent with our prediction that improved spatial perception transfers to separation of signal from noise.

Following convention, speech reception threshold (SRT) was calculated as the SNR that corresponds to the 50% point in the psychometric function fitted for each individual and each noise condition. SRT decreased more in the ILD-train than in the ILD-control group for spatially separated noise (Fig. 2D; rmANOVA, group by test interaction: F_1, 15_ = 13.02, p = 0.003, partial η^2^ = 0.465; effect of test: F_1, 15_ = 8.27, p = 0.012, partial η^2^ = 0.355; effect of group: F_1, 15_ = 0.19, p = 0.666, partial η^2^ = 0.013). Post hoc comparisons revealed that SRT improved in the ILD-train group (p = 0.001), but not in the ILD-control group (p = 0.576), consistent with the pattern of raw identification score and our hypothesis.

We predicted that ILD training would improve speech-in-noise perception by enhancing signal–noise separation using ILD, i.e., ILD-based spatial release from masking (SRM). ILD-based SRM, calculated as SRT difference between spatially separated and collocated conditions (Fig. 2D), was enhanced by ILD training (rmANOVA, group by test interaction: F_1, 16_ = 4.69, p = 0.046, partial η^2^ = 0.227). When calculated as increase in identification score brought about by spatial separation of noise across all SNRs, SRM showed only a trend of ILD-training induced improvement (rmANOVA, group by test interaction: F_1, 54_ = 3.93, p = 0.063, partial η^2^ = 0.179). Between group comparisons revealed that the SRM gain took place primarily at the lower SNRs (Fig. 2E; one-way ANOVA, SNR of − 12 dB: F_1, 18_ = 7.97, p = 0.011, partial η^2^ = 0.307; p > 0.03 for SNR of − 9 dB and p > 0.1 for higher SNRs; alpha was set at 0.013 for correction of multiple comparisons). Moreover, the SRM improvement at SNR of − 12 dB correlated positively with ILD learning at the 6-dB standard location (Fig. 2F; r = 0.519, p = 0.019).

#### ITD training

**Figure 3:**
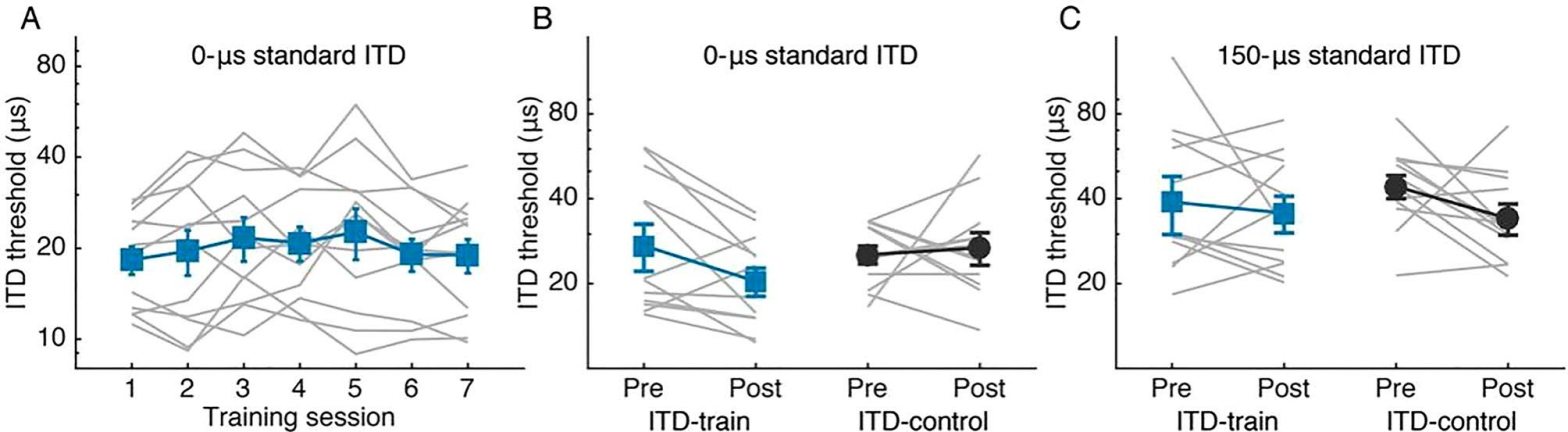
ITD discrimination performance through training. Individual (grey lines) and group mean (filled symbols) ITD discrimination thresholds were plotted through training sessions **(A)** and between the pre- and post-training tests for the training condition **(B)** and an untrained location of 150-μs standard ITD **(C)**.

ITD discrimination was trained with a 1 k-Hz low-pass noise around a standard location of 0-μs ITD (the midline). Unlike ILD training, ITD discrimination threshold did not improve with training (Fig. 3A; rmANOVA, effect of session: F_6, 66_ = 1.74, p = 0.125, partial η^2^ = 0.137; linear regression: F_1,5_ = 0.05, p = 0.832, adjusted R^2^ = 0.01). Between the pre- and posttests, the ITD-train group also performed similarly to untrained controls on both the trained location (Fig. 3B; rmANOVA, group effect: F_1, 22_ = 0.54, p = 0.471, partial η^2^ = 0.024; test effect: F_1, 22_ = 1.61, p = 0.22, partial η^2^ = 0.068; group by test interaction: F_1, 22_ = 3.47, p = 0.076, partial η^2^ = 0.136) and an untrained location of 150-μs ITD (Fig. 3C; group effect: F_1, 22_ = 0.08, p = 0.778, partial η^2^ = 0.004; test effect: F_1, 22_ = 2.72, p = 0.114, partial η^2^ = 0.11; group by test interaction: F_1, 22_ = 0.64, p = 0.432, partial η^2^ = 0.028). The lack of training-induced learning in ITD discrimination was consistent with previous reports^21,37^.

Speech perception in noise was measured in the same task as in the ILD training experiment, except that ITD instead of ILD was varied to lateralize the noise in the spatially separated condition. Also, as the pre-training session in the ILD training experiment produced only a moderate learning effect on the speech task (increase of 3.9 ± 10% in identification score), the pre-training session was skipped in the ITD training experiment.

**Figure 4:**
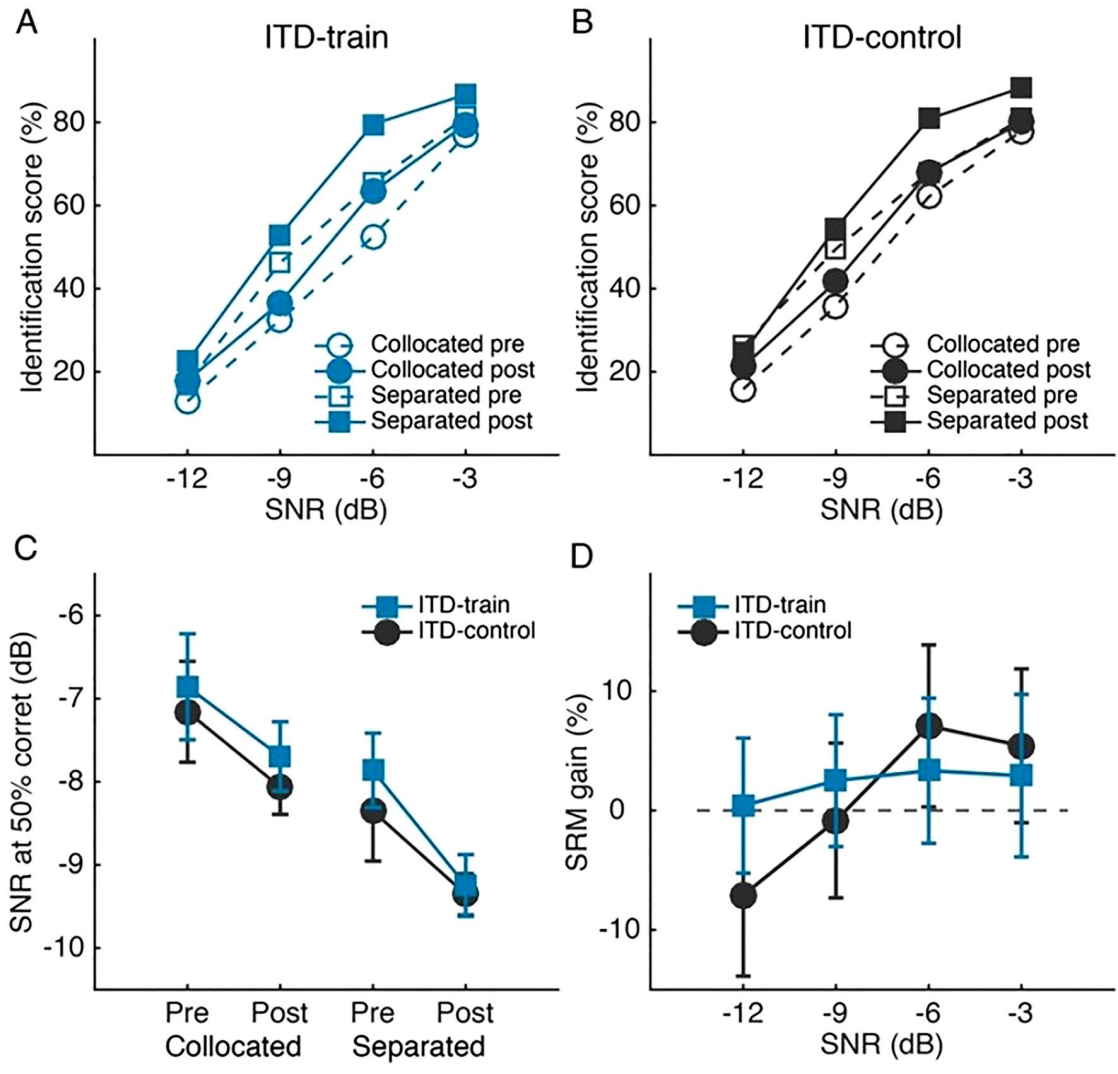
Effect of ITD discrimination training on speech perception in noise. **(A,B)** Mandarin word identification score (in % correct) across SNR levels for the ITD-train **(A)** and ITD-control **(B)** groups. **(C)** Speech reception threshold (SNR at 50% correct identification) changes between pre- and post-training tests. **(D)** Spatial release from masking (SRM) gain in identification score.

ITD training failed to impact speech perception in noise. Before training, similar to the ILD experiment, an ITD-based SRM was observed in both groups (Fig. 4A,B; rmANOVA, effect of condition: F_1, 22_ = 17.49, p < 0.001, partial η^2^ = 0.443; effect of group: F_1, 22_ = 1.195, p = 0.286, partial η^2^ = 0.052; group by condition interaction: F_1, 22_ = 0.02, p = 0.899, partial η^2^ = 0.001). Between the pre- and posttests, the ITD-control (Fig. 4A) and the ITD-train (Fig. 4B) groups improved similarly on identification score (rmANOVA, effect of test: F_1, 22_ = 20.59, p < 0.001, partial η^2^ = 0.483; effect of group: F_1, 22_ = 1.24, p = 0.278, partial η^2^ = 0.053; group by test interaction: F_1, 22_ = 0.28, p = 0.604, partial η^2^ = 0.012; group by test by condition interaction: F_1, 22_ = 0.05, p = 0.824, partial η^2^ = 0.002). Speech reception threshold (SRT) also showed similar pre-to-posttest improvements (Fig. 4C) between the two groups and the two spatial configurations (rmANOVA, effect of test: F_1, 16_ = 10.41, p = 0.05, partial η^2^ = 0.394; effect of group: F_1, 16_ = 0.33, p = 0.577, partial η^2^ = 0.02; all interaction effects: p > 0.4), indicating a nonspecific test–retest effect. Finally, ITD-based SRM (Fig. 4D) did not improve with training (rmANOVA, group effect: F_1, 22_ = 0.05, p = 0.824, partial η^2^ = 0.002; SNR effect: F_3, 66_ = 0.82, p = 0.488, partial η^2^ = 0.036; group by SNR interaction: F_3, 66_ = 0.379, p = 0.768, partial η^2^ = 0.017).

### Training spectral skills

**Figure 5:**
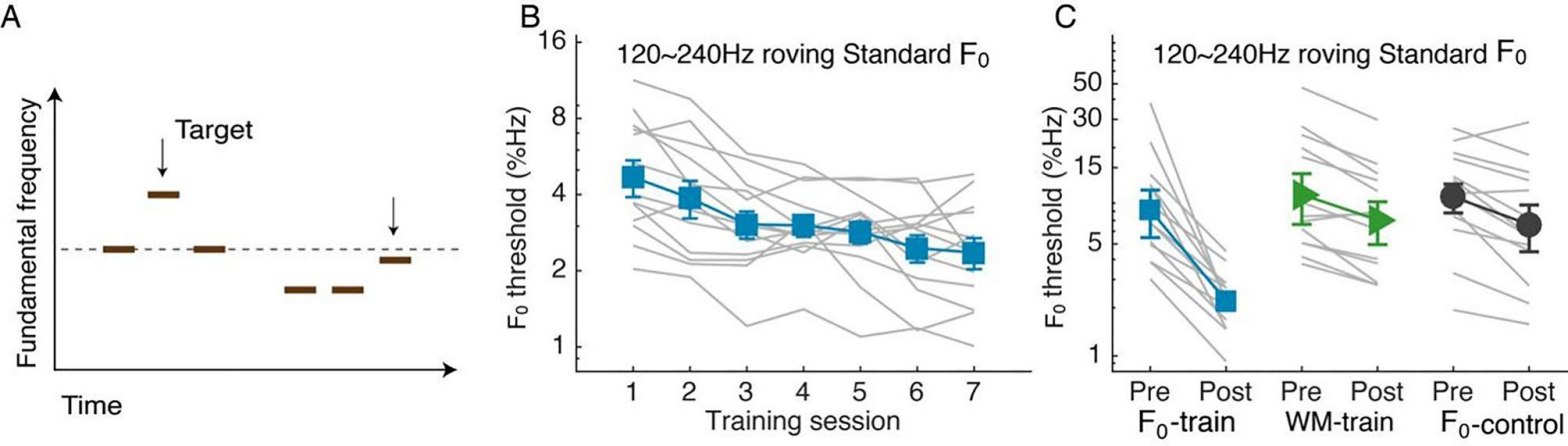
F_0_ discrimination training. **(A)** Illustration of F_0_ discrimination task (for 2 consecutive trials). **(B,C)** Individual (grey lines) and group mean (filled symbols) F_0_ discrimination thresholds through training sessions **(B)** and between the pre- and post-training tests **(C)**.

In the second study, we trained a new group of listeners on F_0_ discrimination (N = 13; Fig. 5A) with high-order (from the 10th to the 20th) harmonic tones. To promote transferable learning, the standard F_0_ was roved between 120 to 240 Hz, approximately equivalent to the range of human voice. According to our hypothesis and previous study^9^, standard frequency roving during frequency discrimination training would engage constant updating of frequency representations in working memory (WM), leading to WM improvement. To control for possible effect of working memory learning, we trained a separate group (N = 13) on Tone n-back, an auditory WM task, Before and after training, the F_0_-train and the WM-train groups as well as an untrained F_0_-control group (N = 13), were tested on the F_0_ task, the WM task, and speech perception in noise.

F_0_ discrimination threshold decreased through the seven training sessions (Fig. 5B; linear regression: F_1,5_ = 60.57, p = 0.001, adjusted R^2^ = 0.909). Consistently, between the pre- and posttests, the F_0_-train group improved more than the WM-train and the F_0_-control groups (Fig. 5C; rmANOVA, group by test interaction: F_2, 36_ = 17.13, p < 0.001, partial η^2^ = 0.488; effect of test: F_1, 36_ = 84.92, p < 0.001, partial η^2^ = 0.702; effect of group: F_2, 36_ = 3.98, p = 0.028, partial η^2^ = 0.181). Interestingly, F_0_ discrimination did not differ between the WM-train and the F_0_-control groups (group by test interaction: F_1, 24_ = 0.06, p = 0.806, partial η^2^ = 0.003), indicating that F_0_ perception, unlike pure-tone pitch perception^9^, did not benefit from WM training.

**Figure 6:**
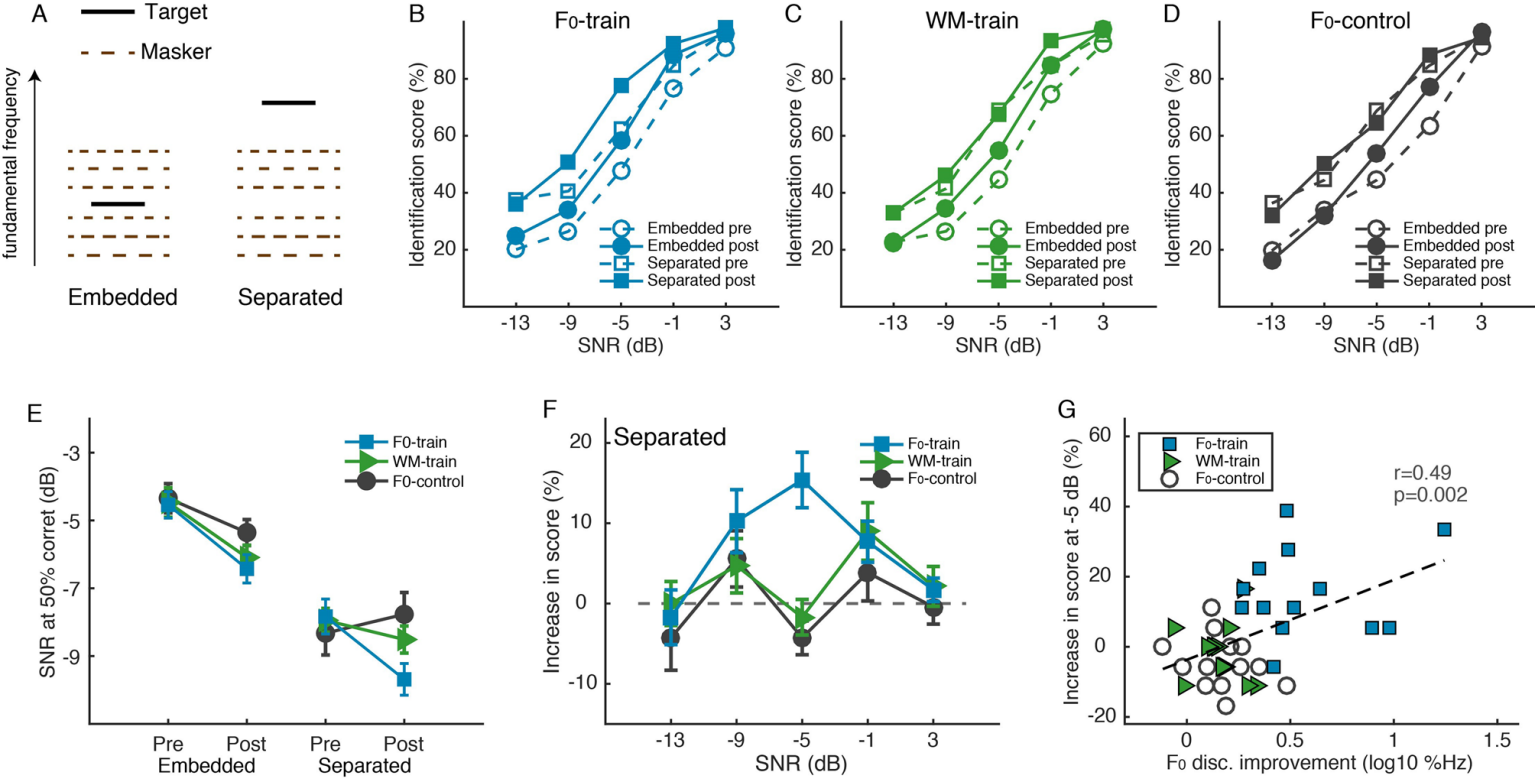
Effect of F_0_ discrimination training on speech perception in noise. **(A)** Illustration of F_0_ relationship of the target and masking speech in the vowel identification task. **(B–D)** Vowel identification score (in % correct) across SNR levels for the F_0_-train **(A)**, WM-train **(B)** and F_0_-control **(C)** groups. **(E) **Speech reception threshold (SNR at 50% correct identification). **(F)** Pre-to-posttest changes of identification score in the spectrally separated condition. **(G)** Correlation between vowel identification improvement at the mid-SNR level (− 5 dB) and F_0_ discrimination learning.

Speech perception in noise was measured by identification of Mandarin vowels spoken by a target speaker in babble noise consisting of mixed speech of six different speakers (Fig. 6A). All masker voices were male with F_0_s between 78 and 161 Hz, equally distributed. The target speaker was either a male with an F_0_ of the mean of the six masker F_0_s (the embedded condition) or a female with an F_0_ well above the masker F_0_ range (the spectrally separated condition). Before training, all of the groups performed better on the spectrally separated than on the embedded condition (Fig. 6B,C,D; rmANOVA, effect of condition: F_1, 36_ = 116.91, p < 0.001, partial η^2^ = 0.765; effect of group: F_2, 36_ = 0.005, p = 0.995, partial η^2^ < 0.002; group by condition interaction: F_2, 36_ = 0.66, p = 0.525, partial η^2^ = 0.035), demonstrating pitch based masking release. Between the pre- and posttests, the three groups improved equally for the embedded condition (rmANOVA, test effect: F_1, 36_ = 40.94, p < 0.001, partial η^2^ = 0.532; group effect: F_2, 36_ = 1.98, p = 0.153, partial η^2^ = 0.099; group by test interaction: F_2, 36_ = 1.10, p = 0.343, partial η^2^ = 0.058), indicating test–retest learning. The spectrally separated condition, however, showed different amounts of learning across groups (group by test interaction: F_2, 36_ = 4.62, p = 0.016, partial η^2^ = 0.204; test effect: F_1, 36_ = 12.92, p = 0.001, partial η^2^ = 0.264; group effect: F_2, 36_ = 0.50, p = 0.612, partial η^2^ = 0.027). Between-group comparisons revealed that the F_0_-train group (group by test interaction: F_1, 24_ = 7.90, p = 0.010, partial η^2^ = 0.248), but not the WM-train group (group by test interaction: F_1, 24_ = 2.34, p = 0.139, partial η^2^ = 0.089), improved more than the F_0_-control group. The group differences were also illustrated with the speech reception threshold (Fig. 6E), which improved equivalently among the three groups on the embedded condition (rmANOVA, group by test interaction: F_2, 31_ = 0.564, p = 0.575, partial η^2^ = 0.035; test effect: F_1, 31_ = 20.04, p < 0.001, partial η^2^ = 0.393; group effect: F_2, 31_ = 1.23, p = 0.306, partial η^2^ = 0.074), but improved more in the F_0_-train group than the other two groups on the spectrally separated condition (group by test interaction: F_2, 28_ = 5.976, p = 0.007, partial η^2^ = 0.299; test effect: F_1, 28_ = 5.04, p = 0.033, partial η^2^ = 0.153; group effect: F_1, 28_ = 0.737, p = 0.487, partial η^2^ = 0.050). A closer examination of performance change on the spectrally separate condition (Fig. 6F) revealed that the additional learning of the F_0_-train group primarily occurred at the mid-SNR level (rmANOVA, group by SNR interaction: F_8, 144_ = 2.348, p = 0.021, partial η^2^ = 0.115; post hoc group comparison, Sidak: p < 0.001 at −5-dB SNR, p > 0.6 at other SNRs). Moreover, performance improvement at this SNR level correlated positively with F_0_ discrimination learning (Fig. 6G; r = 0.49, p = 0.002).

## Discussion

The current results demonstrate that training basic auditory perception, namely discrimination of fine spatial or spectral differences in simple non-speech sounds, can improve speech recognition in noise. In that the trained spatial and spectral cues are used to separate signal from noise, the results support our hypothesis that learning would transfer between tasks involving processes at different levels of information processing. The hypothesis challenges the current view on learning specificity to the training task, suggesting broad existence of learning transfer between tasks. To emphasize the contingence of between-task transfer on their relation, we refer to this hypothesis as *the principle of vertical transfer*. In the literature of perceptual learning, the current results would constitute “far transfer”, as the training and transfer tasks differed categorically in task demand and stimulus type. However, the transfer was not boundless, but displayed a number of limitations or specificities. First, improvement of discrimination performance appears to be a prerequisite for transfer. For similar amount and method of training, while ILD discrimination improved and transferred to speech-in-noise perception, ITD discrimination did not. Though ITD discrimination has been shown to improve with training under some circumstances^23,24^, the lack of ILD comparable training effect was consistent with previous reports^21,37^. In this sense, ITD discrimination training could serve as active control for ILD discrimination training, indicating that the time, exposure, and effort involved in training were insufficient, and that learning of the trained task was necessary, to produce the far transfer. Second, speech perception improved only when noise was separable from target stimuli using the trained spatial or spectral cue, indicating that discrimination learning specifically improved the ability to release noise masking, not speech processing per se. Further, on the separated conditions, transfer was significant only for middle to low signal-to-noise ratios, consistent with the fact that performance benefit of noise separation depends on nature and amount of noise masking^38^. Third, training and learning of auditory working memory did not transfer to speech-in-noise perception (Fig. 6), despite the critical role of working memory suggested for speech recognition^39^. This is probably due to the use of vowel identification for target task, which involved only isolated monosyllables, rendering it unlikely for working memory to become a performance-limiting factor. All taken together, the far transfer from fine discrimination of sound features to speech-in-noise perception is by no means an overthrow of the specificities that have long been observed for perceptual learning, but rather coexists with them. Indeed, the coexisting specificities rather support our hypothesis by demonstrating that between-task transfer occurs only when the proposed contingence is met.

Under the current experimental design, the exact nature of the learned skills, hence the specific mechanisms of their contribution to speech perception in noise, cannot be determined. The training conditions were designed based on previous learning studies^22,34^ to promote the likelihood of learning and across-stimulus transfer, with little effort to limit possibilities of multiple learning mechanisms. For ILD training, ILD was applied to sounds presented through headphones by increasing sound level at one ear and decreasing at the other. Though instructed to indicate change in the lateralized sound image, a listener could perform the discrimination task by listening to sound level change at one ear only and acquire the spatial release for speech-in-noise perception by listening to the ear with better signal-to-noise ratios, namely “better-ear listening” ^12–15^. Spatial separation by an ILD of 6 dB would yield a 3-dB better-ear advantage. The observed spatial release when calculated in SRT (Fig. 2c) was 2.4 dB before and 3.8 dB after training, not much beyond the expected better-ear advantage. Alternative to improving ILD discrimination, ILD training might have improved monaural level discrimination while ignoring input from the other ear, which could have transferred to speech-in-noise perception by improving better-ear listening. Thus, ILD training benefits could be binaural, monaural, or a combination of the two in nature. For F_0_ training, the use of relatively high-order harmonics (10th to 20th order for F_0_ of 120 to 240 Hz) may promote utilization of “temporal fine structure” ^17^, a skill considered by some researchers to be important for speech perception in noise with amplitude fluctuations by allowing for “temporal glimpsing” ^18–20^. On the other hand, in the case of two competing speech stimuli, the contribution of high-order harmonics to masking release was much smaller than low-order ones, particularly for small F_0_ differences^40,41^. Further, the use of multi-talker babble noise, a most effective masker for phoneme stimuli^35,36^, discourages speech segregating mechanisms relying on the masker’s harmonicity such as the harmonic cancellation model^42,43^, but leaves intact other mechanisms such as spectral glimpsing^44,45^. Indeed, it has been suggested that F_0_-difference based speech-masker separation involves a combination of both temporal and spectral mechanisms^46^ and that the pattern and mechanism of masking release depend on the nature of the masker^47^. Thus, the current F_0_ training benefits could be spectral, temporal, or combined in mechanism. For both training experiments, as all candidate skills for learning are also contributing skills for masking release, the uncertainty in learning and transfer mechanisms bears little consequence for our proposal and examination of between-task learning transfer.

While the exact mechanisms of the observed far transfers remain to be specified, there is a straightforward functional link between improved perceptual acuity and reduced noise masking. When discrimination threshold of a sound feature (nominally ILD or F_0_) decreased with training, the perceived distance of a fixed amount of variation in that feature or its associated cues would increase correspondingly, causing greater separation of signal and noise along that perceptual dimension. This idea is supported by the correlations between the threshold decrease for the trained discrimination tasks and the speech intelligibility increase on the speech-in-noise tasks (Figs. 2F; 6G). Alternatively, discrimination training could have led to cognitive changes, such as improved attention control or working memory for better-ear listening in presence of ILD difference or for temporal/spectral glimpsing in presence of F_0_ difference, hence enhancing the utility of that feature in separating noise from signal. The cognitive view, though tempting in its easy accountability for far transfers, is not compatible with the aforementioned specificities coexisting with learning transfer, particularly the lack of transfer from working memory training.

The observed “vertical” transfer between tasks of different levels of complexity and neural processing may be a rule rather than oddity of perceptual learning. Most reports of task specificity have examined transfer between tasks of similar levels, such as feature discrimination along different stimulus dimensions^for review, see^ ^1,5–7^. The critical skills trained with such tasks can be deemed “parallel”, in that they involve information at similar levels of perceptual processing hierarchy that could be computed separately from and independently of each other. In the few cases where non-parallel tasks were examined e.g.,^48,49^ across-task transfer has indeed been reported, with the transfer pattern matching the relation of the tasks in question. For example, training an asynchrony task (whether two tones ended at the same time) transferred to an order task (which tone ended earlier), but not vice versa^48^, which were interpreted as training the two tasks affecting “asymmetric” neural circuits. In another case^49^, learning was reported to transfer between a visual alignment task (whether three elements were aligned) and a bisection task (whether three elements were equally spaced), which was accounted for by the two tasks sharing the same skill (positional judgement along the same spatial axis). Together with our previous report of learning transfer between auditory frequency discrimination and working memory^9^ and the current data, the pattern emerges that learning transfers readily between tasks that are non-parallel, with shared component processes or contributing to each other. That is, perceptual learning is intrinsically capable of “far”, across-task transfer despite its specificity for stimulus and task variation at “near” grounds. While most preceding theories of perceptual learning try to account for learning specificity or transfer in the form of neural modification locus^50^ and/or mechanism^51–54^, the principle of vertical transfer, assuming that auditory performance in most situations involve a shared hierarchical network of sensory, perceptual, affective and cognitive processes organized parallelly at the same level and serially across levels, accounts for learning specificity or transfer in terms of the relationship of the trained process with the processing network of the transfer task^5^. For example, in light of the “learning loci” theories, the current results would be interpreted as learning taking place somewhere “high” along or even beyond the perceptual processing hierarchy, where neurons would respond widely to different stimuli and task demands. In contrast, according to the principle of vertical transfer, learning could take place at relatively low level of sensory processing, befitting the trained sound feature, but is transferrable to “upstream”, more complicated tasks because performance of such tasks would engage the low-level sensory processes. The proposed principle of learning is in line with the multiplexing theory of the auditory system^55^, as well as with a plethora of evidence for rapid, goal oriented plasticity of auditory cortices that allow the same neurons to subserve multiple tasks^56,57^.

On the practical side, the principle of vertical transfer supports broad and effective applications of perceptual learning. Long and much effort has been spent on ways to overcome learning specificities so that perception in challenging environments or challenged populations can benefit from perceptual training^8,58^. Novel training regimens^4,59,60^ have been designed and recreational video games have been exploited^61,62^ to boost learning and its transferability. The current results indicate that the “vertical”, across-task transfers, being far relative to the aims of most previous endeavors, may have been present all the time. Given this principle, an effective way to improve real-life perceptual performance would be training “the shared ground”, i.e., the basic skills most widely involved in target situations of application. The current study, demonstrating that speech perception in noise could benefit from discrimination training of different sound features, provides a first and successful example towards such applications.
